# Long COVID in hospitalized and non-hospitalized patients in a large cohort in Northwest Spain, a prospective cohort study

**DOI:** 10.1038/s41598-022-07414-x

**Published:** 2022-03-01

**Authors:** Alexandre Pérez-González, Alejandro Araújo-Ameijeiras, Alberto Fernández-Villar, Manuel Crespo, Eva Poveda, the Cohort COVID-19 of the Galicia Sur Health Research Institute, Jorge Julio Cabrera, Víctor del Campo, Beatriz Gil de Araujo, Carlos Gómez, Virginia Leiro, María Rebeca Longueira, Ana López-Domínguez, José Ramón Lorenzo, María Marcos, María Teresa Pérez, Lucia Patiño, Sonia Pérez, Silvia Pérez-Fernández, Cristina Ramos, Benito Regueiro, Cristina Retresas, Tania Rivera, Olga Souto, Isabel Taboada, Susana Teijeira, María Torres, Vanesa Val, Irene Viéitez

**Affiliations:** 1 Group of Virology and Pathogenesis, Internal Medicine Department, Álvaro Cunqueiro Hospital, Galicia Sur Health Research Institute (IIS Galicia Sur), Complexo Hospitalario Universitario de Vigo, SERGAS-UVigo, Vigo. St. Clara Campoamor Nº 341, 36312 Vigo, Spain; 2 Infectious Diseases Unit, Department of Internal Medicine, Galicia Sur Health Research Institute (IIS Galicia Sur), Complexo Hospitalario Universitario de Vigo, SERGAS-UVigo, Vigo, Spain; 3 Pneumology Service, Galicia Sur Health Research Institute (IIS Galicia Sur), Complexo Hospitalario Universitario de Vigo, SERGAS-UVigo, Vigo, Spain; 4 Infectious Diseases Department, Hospital Universitari de Bellvitge, IDIBEL, Barcelona, Spain; 5 Galicia Sur Health Research Institute (IIS Galicia Sur), Vigo, Spain

**Keywords:** Infectious diseases, Respiratory tract diseases

## Abstract

Survivors to COVID-19 have described long-term symptoms after acute disease. These signs constitute a heterogeneous group named *long COVID* or *persistent COVID*. The aim of this study is to describe persisting symptoms 6 months after COVID-19 diagnosis in a prospective cohort in the Northwest Spain. This is a prospective cohort study performed in the COHVID-GS. This cohort includes patients in clinical follow-up in a health area of 569,534 inhabitants after SARS-CoV-2/COVID-19 diagnosis. Clinical and epidemiological characteristics were collected during the follow up. A total of 248 patients completed 6 months follow-up, 176 (69.4%) required hospitalization and 29 (10.2%) of them needed critical care. At 6 months, 119 (48.0%) patients described one or more persisting symptoms. The most prevalent were: extra-thoracic symptoms (39.1%), chest symptoms (27%), dyspnoea (20.6%), and fatigue (16.1%). These symptoms were more common in hospitalized patients (52.3% vs. 38.2%) and in women (59.0% vs. 40.5%). The multivariate analysis identified COPD, women gender and tobacco consumption as risk factors for long COVID. Persisting symptoms are common after COVID-19 especially in hospitalized patients compared to outpatients (52.3% vs. 38.2%). Based on these findings, special attention and clinical follow-up after acute SARS-CoV-2 infection should be provided for hospitalized patients with previous lung diseases, tobacco consumption, and women.

## Introduction

COVID-19 pandemic has been a major health challenge around the world, with an unprecedented impact on health systems, society and modern medicine. Acute phase of COVID-19 varies widely, from asymptomatic disease to pneumonia, acute respiratory distress syndrome (ARDS) and multisystem organ failure. Most commonly reported symptoms are fever, cough, dyspnoea, myalgia and anosmia. Several comorbidities have been identified as risk factors for severe disease, such as arterial hypertension, chronic heart disease or diabetes mellitus^1^.

In the past few months, many patients reported persisting symptoms after the acute phase of coronavirus disease. These signs are a heterogeneous group, affecting multiple organ systems, including respiratory tract (dyspnoea, chest pain, cough), muscle and joints (arthralgia, myalgia), nervous system (taste or smell alterations, headache, concentration difficulty). There are also many general or unspecified symptoms such as fatigue or hair loss. This group of signs has been named “long COVID” or “persisting COVID”. However, the definition of this syndrome remains unclear and there is not consensus regarding the name and the diagnostic criteria^2^. It is not clear which symptoms could be related to coronavirus and how to define this persistent period after the acute phase^3,4^. The origin of these symptoms remains unknown. However, it is likely to be a combination of a direct damage caused by SARS-CoV-2, previous comorbidities, immunological activation, psychological and emotional factors^5–7^. The aim of this study is to describe persisting symptoms 6 months after COVID-19 diagnosis in a prospective cohort in the Northwest Spain. The study population includes mild, moderate, serious or critical cases classified as hospitalized or non-hospitalized patients.

## Methods

### Study design

This is a prospective cohort study performed in the COVID-19 Cohort of Galicia Sur Health Institute (COHVID-GS). This is an open cohort associated to a Biobank for sample collection including patients in clinical follow-up at Álvaro Cunqueiro Hospital (Vigo, Spain), a reference hospital for 564,534 inhabitants after SARS-CoV-2/COVID-19 diagnosis (all had a positive PCR for SARS-CoV-2 on nasal swab) (https://www.iisgaliciasur.es/apoyo-a-la-investigacion/cohorte-covid19/). Clinical and epidemiological characteristics were collected at the diagnosis time and during the follow up after 6 months of the diagnosis. The aim of this study is to describe the frequency of long COVID symptoms in hospitalized and non-hospitalized subjects.

### Procedures

All patients included at the COHVID-GS were scheduled for clinical follow-up visits using phone calls and on site visits after 1, 3 and 6 months of the diagnosis. Subjects were classified as hospitalized when they required hospitalization for at least 24 h. Main hospitalization criteria were pneumonia on chest X-ray and/or respiratory insufficiency. At each visit, patients completed a custom symptom questionnaire (supplementary data). COHVID-GS also includes data about comorbidities, smoke status, weight and height. Pre-existing symptoms that did not change after the diagnosis of COVID-19 were excluded. Symptoms of another suspected origin were also excluded (e.g., iron deficit, thyroid dysfunction). Dyspnoea was classified according to the British Medical Research Council mMRC scale. Brain fog was defined as poor concentration, recent memory disorder or inability to focus. COPD was defined according to the Global Strategy for the Diagnosis, Management, and Prevention of Chronic Obstructive Pulmonary Disease (GOLD) as a FEV1/FVC < 70% with or without symptoms (cough, sputum production).

In addition, the COHVID-GS records data about several biomarkers during the follow-up. C-reactive protein (CRP; normal range 0.0–8.0 mg/L), ferritin (normal range 22.0–300.0 ng/mL) and lactate dehydrogenase (LDH; normal range 85.0–240.0 IU/L) after 6 months.

In this study, we only included patients who survived at least 6 months after the COVID-19 diagnosis and completed all follow-up visits. We excluded patients who died during the study period, those who refused to complete all follow-up visits and those who were unable to assist to the site (e.g., cognitive impairment, physical disability).

The recruitment period started in March 2020 and ended by May 2021 and included consecutive cases. For the purpose of this study, data was censored in January 2021 when the first wave cases reached 6 months since COVID-19 diagnosis.

### Ethics

All procedures performed in studies involving human participants were in accordance with the ethical standards of the institutional research committee and with the 1964 Helsinki declaration and its later amendments or comparable ethical standards. This project was approved by the Ethical Committee of Pontevedra-Vigo-Ourense (reference 2021/184). All the patients included in the study belong to the COHVID-GS, which signed an informed consent form at the time of inclusion in the cohort.

### Statistical analysis

For quantitative variables, median and interquartile were calculated. Categorical variables were reported as frequencies and percentages. Subjects were classified in two groups, based on their need for hospitalization due to Covid-19. Symptoms, clinical and epidemiological data were compared in both groups using chi-square test, Fisher’s exact test or U Man–Whitney test as appropriate. *p*-value less than 0.05 was considered significant. Multivariable logistic regression analysis with backward stepwise elimination was used to identify risk factors for long COVID. Those variables with a *p* value lesser than 0.1 were considered as independent variables. Statistical analyses were performed with IBM Statistical Product and Service Solutions (SPSS) program, version 22.

## Results

### Baseline characteristics of the study population

A total of 630 patients were included in the COHVID-GS. However, 81 of them died during the hospitalization period and only 248 subjects who survived completed the 6 months period of follow-up. Of them, 172 required hospitalization and 76 were never hospitalized. The baseline characteristics of the study population are shown in Table 1.

**Table 1:** Baseline characteristics of study population.

	Total	Hospitalized	Non hospitalized	*p*-value
Patients in follow-up, n (%)	248 (100%)	172 (69.4%)	76 (30.6%)	
**Demographics**				
Age, in years, median (IQR)	57 (46–68)	62 (51–71)	47 (34–54)	*p* < 0.001
Sex, men, n (%)	148 (59.7%)	103 (59.9%)	45 (62.5%)	*p* = 1.000
Spanish, n (%)	234 (94.4%)	159 (92.4%)	75 (98.7%)	*p* = 1.000
Latin American, n (%)	11 (4.4%)	10 (5.8%)	1 (1.3%)	*p* = 0.070
European (not Spanish), n (%)	2 (3.8%)	2 (1.2%)	0	*p* = 0.267
Oceania, n (%)	1 (0.4%)	1 (0.6%)	0	*p* = 1.000
**Severity**				
Admission to critical care, n (%)	29 (11.7%)	29 (16.8%)	N/A	N/A
Invasive mechanical ventilation required, n (%)	23 (9.3%)	23 (13.4%)	N/A	N/A
**Comorbidities**				
Obesity, n (%)	75 (30.2%)	62 (36.0%)	13 (17.1%)	*p* < 0.001
Arterial hypertension, n (%)	65 (26.2%)	58 (33.7%)	7 (9.2%)	*p* < 0.001
Diabetes mellitus, n (%)	26 (10.5%)	25 (14.5%)	1 (1.3%)	*p* = 0.001
Asthma, n (%)	21 (8.5%)	7 (4.1%)	14 (18.4%)	*p* = 0.807
Chronic ischaemic heart disease, n (%)	21 (8.5%)	21 (12.2%)	0	*p* < 0.001
Chronic obstructive pulmonary disease, n (%)	15 (6.0%)	15 (8.7%)	0	*p* = 0.007
Active cancer, n (%)	5 (2.0%)	4 (2.3%)	1 (1.3%)	*p* = 1.000
HIV infection, n (%)	3 (1.2%)	2 (1.2%)	1 (1.3%)	*p* = 1.000
Chronic kidney disease, n (%)	3 (1.2%)	3 (1.7%)	0	*p* = 0.555
Liver cirrhosis, n (%)	2 (0.8%)	2 (1.2%)	0	*p* = 1.000
**Tobacco use**				
Active smoker, n (%)	18 (7.3%)	8 (4.7%)	10 (13.1%)	*p* = 0.031
Former smoker, n (%)	105 (42.3%)	75 (43.6%)	30 (39.5%)	*p* = 0.575
Never smoker, n (%)	120 (48.4%)	85 (49.4%)	35 (46.1%)	*p* = 0.582
Missing information, n (%)	5 (2.0%)	4 (2.3%)	1 (1.3%)	
Total	248 (100%)	172 (69.4%)	76 (30.6%)	

Overall, the median age was 57 years, however, non-hospitalized patients were significantly younger than those who were hospitalized (62 vs. 47 years, respectively). Moreover, men were more frequent in both groups (148 men, 59.7%).

Most common comorbidities were obesity (n = 75, 30.2%), followed by arterial hypertension (n = 65, 26.2%) and diabetes mellitus (n = 26, 10.1%). Moreover, all comorbidities were more usual in the hospitalized group, except for asthma (4.1% vs. 18.4% respectively). In the hospitalized group, 29 patients (16.8%) required critical care management, including 23 who required invasive mechanical ventilation.

6 months after the COVID-19 diagnosis, 119 patients (48.9%) reported at least one symptom (Table 2). Chest symptoms were more frequent (n = 67, 27%), of which dyspnoea (n = 51, 20.6%), chest pain (n = 15, 6.0%) and cough (n = 11, 4.4%) were the most commonly reported.

**Table 2:** Prevalence of 6 months persisting symptoms and serum biomarkers.

	Total	Hospitalized	Non hospitalized	*p*-value
Total patients, n (%)	248 (100%)	172 (69.4%)	76 (30.6%)	
Any symptom, n (%)	119 (48.0%)	90 (52.3%)	29 (38.2%)	*p* = 0.040
Chest symptoms, n (%)	67 (27.0%)	55 (32.0%)	12 (15.8%)	*p* = 0.008
Dyspnoea, n (%)	51 (20.6%)	43 (25.0%)	8 (10.5%)	*p* = 0.010
Grade MMRC 1, n (%)	43 (17.3%)	36 (20.9%)	7 (0.9%)	*p* = 0.029
Grade MMRC 2, n (%)	8 (3.2%)	7 (4.1%)	1 (1.3%)	*p* = 0.441
Chest pain, n (%)	15 (6.0%)	11 (6.4%)	4 (5.3%)	*p* = 0.444
Cough, n (%)	11 (4.4%)	10 (5.8%)	1 (1.3%)	*p* = 0.673
Extra-thoracic symptoms, n (%)	97 (39.1%)	74 (43.0%)	23 (30.3%)	*p* = 0.058
Digestive symptoms, n (%)	10 (4.0%)	7 (4.1%)	3 (3.9%)	*p* = 0.999
Abdominal pain, n (%)	7 (2.8%)	5 (2.9%)	2 (2.6%)	*p* = 1.000
Diarrhoea, n (%)	4 (1.6%)	3 (1.7%)	1 (1.3%)	*p* = 1.000
Musculoskeletal symptoms, n (%)	18 (7.3%)	16 (9.3%)	2 (2.6%)	*p* = 0.068
Myalgia, n (%)	10 (4.0%)	10 (5.8%)	0	*p* = 0.034
Arthralgia, n (%)	12 (4.8%)	10 (5.8%)	2 (2.6%)	*p* = 0.354
Fatigue, n (%)	40 (16.1%)	36 (20.9%)	4 (5.3%)	*p* = 0.001
Anosmia, n (%)	17 (6.9%)	9 (5.2%)	8 (10.5%)	*p* = 0.054
Ageusia, n (%)	10 (4.0%)	5 (2.9%)	5 (6.6%)	*p* = 0.181
Headache, n (%)	12 (4.8%)	6 (3.5%)	6 (8.0%)	*p* = 0.196
Brain fog, n (%)	9 (3.6%)	9 (5.2%)	0	*p* = 0.061
Mood disorder, n (%)	10 (4.0%)	5 (2.9%)	5 (6.6%)	*p* = 0.396
Hair loss, n (%)	18 (7.3%)	16 (9.3%)	2 (2.6%)	*p* = 0.068
Sleep disorder, n (%)	9 (3.6%)	9 (5.2%)	0	*p* = 0.061
Conjunctival chemosis, n (%)	1 (0.4%)	1 (0.6%)	0	*p* = 1.000
**Serum biomarkers**				
CRP, median (mg/dL), IQR	0.93 (2.83)	1.19 (3.40)	0.35 (1.70)	*p* = 0.001
LDH, median (IU/L), IQR	189.0 (43.0)	176.5 (33.0)	192.0 (41.0)	*p* < 0.001
Ferritin, median (ng/mL), IQR	78.0 (108.0)	82.0 (107.0)	74.0 (109.0)	*p* = 0.486

*CRP* C-reactive protein, *LDH* lactate dehydrogenase, *IQR* interquartile range.

Extra-thoracic symptoms were described in 97 patients (39.1%) and the most frequently reported were fatigue (n = 40, 16.1%), musculo-skeletal symptoms (n = 18, 7.3%) and hair loss (n = 18, 7.3%).

Overall, smell disorders were more frequent than taste disorders (6.9% vs. 4.0%, respectively). Neurological symptoms 6 months after the COVID-19 diagnosis, excluding smell and taste disruptions, were uncommon (less than 5.0%). Headache, mood disorder and sleep disturbance were the most common neurological symptoms. Nine patients (3.2%) reported symptoms that met *brain fog* criteria. Digestive symptoms were also uncommon (n = 10, 4.0%).

### Persisting symptoms in hospitalized and non-hospitalized patients

Persisting symptoms were more prevalent in the hospitalized group (52.3% vs. 38.2%, *p* = 0.040). Chest symptoms in this group were more frequently reported compared with the non-hospitalized group (32.0% vs. 15.8%, *p* = 0.008). However, extra-thoracic symptoms did not differ between both groups (43.0% vs. 30.3%, *p* = 0.058). The most common chest symptom was dyspnoea, which was more frequently reported in hospitalized patients (25.0% vs. 10.5%, *p* = 0.010). Most of the subjects reported a mMRC (modified Medical Research Council) grade 1 dyspnoea (n = 43, 17.3%). On the other hand, chest pain (6.4% vs. 5.3%) and persisting cough (5.8% vs. 1.3%) did not vary between both groups.

Fatigue (20.9% vs. 5.3%, *p* = 0.001) and myalgia (5.8% vs. 0%, *p* = 0.034) were more frequently reported in the hospitalized group than in the non-hospitalized group. Also, hair loss (9.3% vs. 2.6%) and sleep disorders (5.2% vs. 0%) were more common in hospitalized patients, although this difference was not statistically significant. Arthralgia (5.8% vs. 2.6%), ageusia (2.9% vs. 6.6%) and digestive symptoms (4.1% vs. 3.9%) did not differ between both groups. Anosmia was more prevalent in non-hospitalized subjects (5.2% vs. 10.5%, *p* = 0.054), but did not meet statistical significance.

### Persisting symptoms in ICU-admitted patients

Persisting symptoms were compared between patients admitted to ICU and patients admitted to the hospital ward. We did not find any association between patients admitted to ICU or to the hospital ward and the persistence of any symptoms at 6 months.

### Persisting symptoms in men and woman

Overall, persisting symptoms were more common in women than men (59.0% vs. 40.5%, *p* = 0.004) (Table 3). Dyspnoea (29.0 vs. 14.9%, *p* = 0.007), headache (10.0% vs. 4.8%, *p* = 0.004), fatigue (22.0% vs. 12.2%, *p* = 0.039) and hair loss (15.0% vs. 7.3%, *p* < 0.001) were all more prevalent in women while anosmia (6.0% vs. 7.4%), taste disturbance (4.0% in both genders) and musculoskeletal symptoms (6.8% vs. 8.0%) did not differ between women and men.

**Table 3:** Prevalence of persisting symptoms at 6 months based on gender.

	Women	Men	*p*-value
Any symptom, n (%)	59 (59.0%)	60 (40.5%)	*p* = 0.004
Chest symptoms, n (%)	35 (35.0%)	32 (21.6%)	*p* = 0.020
Dyspnoea, n (%)	29 (29.0%)	22 (14.9%)	*p* = 0.007
Chest pain, n (%)	7 (7.0%)	8 (5.4%)	*p* = 0.600
Cough, n (%)	7 (7.0%)	4 (2.7%)	*p* = 0.125
Extra-thoracic symptoms, n (%)	50 (50.0%)	47 (31.8%)	*p* = 0.004
Neurological symptoms, n (%)	26 (26%)	21 (14.2%)	*p* = 0.022
Anosmia, n (%)	6 (6.0%)	11 (7.4%)	*p* = 0.800
Agueusia, n (%)	4 (4.0%)	10 (4%)	*p* = 1.000
Headache, n (%)	10 (10%)	12 (4.8%)	*p* = 0.004
Brain fog, n (%)	5 (5%)	4 (2.7%)	*p* = 0.491
Diarrhea, n (%)	4 (4.0%)	0	*p* = 0.025
Myalgia, n (%)	5 (5.0%)	5 (3.4%)	*p* = 0.530
Arthralgia, n (%)	4 (4.0%)	8 (5.4%)	*p* = 0.767
Fatigue, n (%)	22 (22%)	18 (12.2%)	*p* = 0.039
Hair loss, n (%)	15 (15%)	18 (7.3%)	*p* < 0.001

### Predictors of COVID-19 persisting symptoms

We performed a multivariate analysis in order to determine the risk factors for persisting COVID-19 symptoms. We identified Chronic Pulmonary Obstructive Disease (COPD) (OR 5.009; CI 95%, 1.321–18.997, *p* = 0.018), woman gender (OR 2.700, 95% CI 1.543–4.724, *p* < 0.001) and tobacco consumption as independent risk factors for remaining symptoms (OR 1.736; CI 95%, 1.002–3.007, *p* = 0.049) (Table 4).

**Table 4:** Risk factors for long COVID.

	Odds Ratio	Confidence interval (95%)	*p*-value
COPD	5.009	1.321–18.997	*p* = 0.018
Sex, women	2.700	1.543–4.724	*p* < 0.001
Hospitalization	1.711	0.951–3.078	*p* = 0.073
Tobbacco consumption	1.736	1.002–3.007	*p* = 0.049

*COPD* chronic obstructive pulmonary disease.

COPD was the only comorbidity associated with a higher prevalence of persisting symptoms. Fourteen patients (5.1%) were affected by COPD before COVID-19 diagnosis and 12 of them (80.0%) had at least one persisting symptom. Nine subjects (75.0%) reported new or worsened dyspnoea compared with their perception prior COVID-19 diagnosis. Dyspnoea (60.0% vs. 18.0%, *p* = 0.001) and chest pain (26.7% vs. 4.7%, *p* = 0.008) were most commonly reported in COPD group. On the other hand, cough (6.7% vs. 4.3%), fatigue (20.0% vs. 15.9%), myalgia (13.3% vs. 3.4%), arthralgia (6.7% vs. 4.7%) did not differ between COPD and non-COPD subjects.

### Biomarkers in symptomatic and asymptomatic patients

The following serum inflammatory biomarkers CRP, ferritin and LDH were assessed 6 months since COVID-19 diagnosis in symptomatic and asymptomatic patients.

Although CRP and LDH levels were higher in the hospitalized group, most of samples met the normal range criteria. Therefore, the levels of inflammatory biomarkers were not associated with the persistence of symptoms.

## Discussion

We characterized long COVID in a prospective cohort of patients with mild and severe COVID-19 with a systematic clinical follow-up. The prevalence of any symptom at 6 months was elevated (48%), with a different distribution between hospitalized and non-hospitalized patients. Persisting symptoms did not differ between those patients who needed critical care and those who did not.

Long COVID has been recognized as a public-health problem and a matter of concern for COVID-19 surveys. Several studies have reported information in series of patients in clinical follow-up after the acute infection, but many questions remain unsolved. One of them is if long COVID might be gender-dependent. In our study, women showed a higher prevalence of persisting symptoms in hospitalized and in non-hospitalized groups. Dyspnoea, headache, fatigue and hair loss were all more common in women than men.

A prior study performed by Huang et al. described a high prevalence of long COVID symptoms in a large cohort of hospitalized patients^8^, around 76%, slightly above our study. The most commonly reported symptoms were fatigue, chronic pain, and anxiety or depression. The prevalence of persisting symptoms was closely related to COVID-19 severity. Our study reported similar symptoms, with a slightly different distribution since we have included an inpatients population, while Huang et al., only included hospitalized patients. This could explain the higher prevalence observed in our series for some symptoms (i.e. dyspnea). Interestingly, they also observed a higher prevalence of long COVID among women compared with men^8^. A recent study by Blomberg et al. also reported a higher frequency of persisting symptoms in hospitalized patients. Similarly to our results, COPD and asthma were also related to a worse fatigue score and a higher number of persisting symptoms^9^. Our findings, reinforces the relationship between persisting symptoms, hospitalization, woman gender, and COPD.

In the recent months, more than 200 persistent symptoms have been described after the acute phase of COVID-19. Most of them are related to the respiratory tract (dyspnoea, cough, chest pain), the main target of SARS-CoV-2. However, many patients report other type of symptoms such as fatigue, cognitive impairment or smell disorders^10^.

Chest symptoms were the most common reported affection, with greater prevalence in the hospitalized group. This difference may be related to pneumonia, respiratory distress and lung damage, which were more frequent in hospitalized subjects. The prevalence of chest symptoms in this group raised to 52%, slightly higher than observed in previous reports^11^. However, even in the absence of pneumonia, 15% of non-hospitalized patients reported persisting chest symptoms. Recently, Blomberg et al., described long COVID symptoms in Norwegian non-hospitalized and hospitalized population with a slightly higher prevalence than in our study (52.0% vs. 38.2% respectively)^9^.

Fatigue was also frequent, reaching up to 20.9% in the hospitalized group. Although this percentage is relevant, it was lower than others reported before^12,13^, around 65–70%. However, these studies had a shorter follow-up period (90–100 days after hospitalization). The reason of fatigue remains unknown. Several causes have been proposed such as direct nervous and muscle damage due to SARS-CoV-2 replication^5,6^, persisting inflammation, or emotional factors^7^.

On the other hand, myalgia and arthralgia were less frequent than in previous studies. Sykes et al.^14^ described a prevalence of 50% of these symptoms in hospitalized patients at 100 days after discharge.

Overall, persisting anosmia was reported in 6.8% of subjects and was more common in the non-hospitalized group (10.5% vs. 5.2%, respectively). This prevalence is lower than published in previous studies^15,16^. In addition, prior reports have suggested that anosmia may be associated with a mild to moderate COVID-19 and it could be more common in women than men^15^. However, the low prevalence of anosmia observed in our study cannot confirm this hypothesis.

At 6 months, 9 subjects reported unspecified neurological symptoms such as poor concentration, loss of recent memory or inability to focus. These unspecified symptoms are named *brain fog* by some authors, but its origin or relationship with coronavirus disease remains unknown^5,17^. *Brain fog* syndrome after COVID-19 has been reported previously^18,19^, although there is not a validated diagnostic criteria established. The studies focused on neurological *sequelae* of COVID-19 did not use a unanimous criterion and therefore, the conclusions are very heterogeneous. The lack of knowledge about this type of symptoms makes more difficult to study the real prevalence or to establish a relationship with coronavirus disease. Another neurological symptoms like sleep or mood disorders have been reported with a similar prevalence than in our study^20^. However, psychological conditions, stress and isolation may be associated with these symptoms, alongside with coronavirus infection. The higher prevalence on the hospitalized group may be due to the severity of illness, admission to critical care or treatments (e.g., corticosteroids, immunosuppressants).

Regarding the impact of ICU admission in the development of persisting symptoms, contradictory results have been reported up to now^21,22^. In our study, we did not find any relationship between ICU admission and the persistence of symptoms. However, the small sample size of ICU-admitted patients could be underestimating this association.

We identified COPD, women gender, tobacco consumption and the need for hospitalization as predictors for long COVID. Women gender has been reported before as a risk factor for hospitalized subjects but there is a lack of information about other predictors of long COVID.

On the other hand, only few studies have evaluated classical inflammatory biomarkers (i.e., CRP, ferritin) in patients with persistent COVID. Pasini et al.^23^ have observed higher levels of CRP, interleukin-6 and soluble CD25 in hospitalized patients 75 days after the COVID-19 diagnosis. However, we did not find any association of CRP, ferritin and LDH with the persistence of symptoms.

Our findings suggest that some of the persisting symptoms could be related to a previous lung comorbidity and hospitalization. In addition, pneumonia and ARDS may also contribute to develop persisting symptoms.

This study has few limitations. Firstly, some of the symptoms reported are subjective and based on the patient’s testimony (e.g. fatigue, headache, dyspnoea). Additionally, the lack of validated scales to measure most of the symptoms makes difficult to compare data between subjects or studies. Secondly, most of the symptoms may be affected by personal, psychological or environmental factors. We needed to use a non-validated custom questionnaire, as there is not a specific post-COVID-19 document established. Many symptoms could be higher reported in one group than another for unrelated reasons with coronavirus disease. This is the case for hair loss that may be more noticed in women and therefore more reported, leading to a possible increase of the prevalence in women. In addition, previous comorbidities and age could increase the persisting symptoms in the hospitalized group. For COPD patients, we did not record symptoms that started before COVID-19 diagnosis. Therefore, it is possible that COPD patients reported more frequently chest or respiratory symptoms.

## Conclusions

In summary, a high prevalence of long COVID (48%) was observed in patients after 6 months of acute SARS-CoV-2 infection. Overall, persistent symptoms were more common in hospitalized patients compared to outpatients (52.3% vs 38.2%). These symptoms were more common in women than men (59.0% vs. 40.5%). COPD, woman gender, and tobacco consumption were identified as long COVID predictors. These findings suggest that in those patients that required hospitalization after SARS-CoV-2 infection a close clinical follow-up might be recommended. Special attention should be provided for patients with previous lung diseases, tobacco consumption and women.

## Supplementary Information

Supplementary Information.

## Data Availability

The datasets during and/or analyzed during the current study available from the **COHVID-GS** on reasonable request (https://www.iisgaliciasur.es/apoyo-a-la-investigacion/cohorte-covid19/).
