# Author Correction: Long COVID in hospitalized and non-hospitalized patients in a large cohort in Northwest Spain, a prospective cohort study

**DOI:** 10.1038/s41598-022-18023-z

**Published:** 2022-08-16

**Authors:** Alexandre Pérez-González, Alejandro Araújo-Ameijeiras, Alberto Fernández-Villar, Manuel Crespo, Eva Poveda, the Cohort COVID-19 of the Galicia Sur Health Research Institute, Jorge Julio Cabrera, Víctor del Campo, Beatriz Gil de Araujo, Carlos Gómez, Virginia Leiro, María Rebeca Longueira, Ana López-Domínguez, José Ramón Lorenzo, María Marcos, María Teresa Pérez, Lucia Patiño, Sonia Pérez, Silvia Pérez-Fernández, Cristina Ramos, Benito Regueiro, Cristina Retresas, Tania Rivera, Olga Souto, Isabel Taboada, Susana Teijeira, María Torres, Vanesa Val, Irene Viéitez

**Affiliations:** 1 Group of Virology and Pathogenesis, Internal Medicine Department, Álvaro Cunqueiro Hospital, Galicia Sur Health Research Institute (IIS Galicia Sur), Complexo Hospitalario Universitario de Vigo, SERGAS-UVigo, Vigo. St. Clara Campoamor Nº 341, 36312 Vigo, Spain; 2 Infectious Diseases Unit, Department of Internal Medicine, Galicia Sur Health Research Institute (IIS Galicia Sur), Complexo Hospitalario Universitario de Vigo, SERGAS-UVigo, Vigo, Spain; 3 Pneumology Service, Galicia Sur Health Research Institute (IIS Galicia Sur), Complexo Hospitalario Universitario de Vigo, SERGAS-UVigo, Vigo, Spain; 4 Infectious Diseases Department, Hospital Universitari de Bellvitge, IDIBEL, Barcelona, Spain; 5 Galicia Sur Health Research Institute (IIS Galicia Sur), Vigo, Spain

Correction to: *Scientific Reports* 10.1038/s41598-022-07414-x, published online 01 March 2022

The original version of this Article contained an error in Table 1, where the values given for the Tobacco use were incorrect for “Total”, “Hospitalized” and “Non Hospitalized”. The correct and incorrect values appear below.

Incorrect:

**Table 1:** Baseline characteristics of study population.

Tobacco use	Total	Hospitalized	Non Hospitalized	*p*-value
Active smoker, n (%)	18 (7.3%)	8 (4.6%)	10 (13.9%)	*p* = 0.031
Former smoker, n (%)	105 (42.3%)	75 (43.6%)	35 (48.6%)	*p* = 0.575
Never smoker, n (%)	120 (69.8%)	85 (49.4%)	35 (20.3%)	*p* = 0.582

Correct:

**Table 1:** Baseline characteristics of study population.

Tobacco use	Total	Hospitalized	Non Hospitalized	*p*-value
Active smoker, n (%)	18 (7.3%)	8 (4.7%)	10 (13.1%)	*p* = 0.031
Former smoker, n (%)	105 (42.3%)	75 (43.6%)	30 (39.5%)	*p* = 0.575
Never smoker, n (%)	120 (48.4%)	85 (49.4%)	35 (46.1%)	*p* = 0.582
Missing information, n (%)	5 (2.0%)	4 (2.3%)	1 (1.3%)	
Total	248 (100%)	172 (69.4%)	76 (30.6%)	

The original Article has been corrected.

