# Supplementary material for: Long COVID in hospitalized and non-hospitalized patients in a large cohort in Northwest Spain, a prospective cohort study
Source: Sci Rep. 2022 Mar 1;12:3369. doi: 10.1038/s41598-022-07414-x (PMC8888560; doi:10.1038/s41598-022-07414-x)
Supplement: Supplementary file 1 — Supplementary Information. [file 41598_2022_7414_MOESM1_ESM.docx]

**SUPPLEMENTARY DATA**

**Questionnaire of follow-up after COVID-19 disease**

- **Blood type**:

A+ A- B+ B- AB+ AB- 0+ 0-

- **Evaluation corresponding to**:

1st Month 3rd Month 6th Month 12th Month Another date:

- **Persistence of symptoms**:
  - No
  - Yes
    - Type of symptoms:
      - **General**

Fatigue Sore throat Hair loss Insomnia

- - - - **Neurologic**:

Loss of smell Loss of taste Headache Tingling Numbness Dizziness Confusion

- - - - **Eye**:

Conjunctivitis Dry eye Chemosis Epiphora

- - - - **Thoracic**:

Cough Chest pain

Dyspnoea → Grade (mMRC Scale): 1 2 3 4

- - - - **Digestive**:

Diarrhoea Nausea Constipation Abdominal pain

- - - - **Musculoskeletal**:

Muscle pain Joint pain
